# The GINA asthma strategy report: what’s new for primary care?

**DOI:** 10.1038/npjpcrm.2015.50

**Published:** 2015-07-30

**Authors:** Helen K Reddel, Mark L Levy

**Affiliations:** 1 Clinical Management Group, Woolcock Institute of Medical Research, University of Sydney, Glebe, NSW, Australia; 2 Kenton Bridge Medical Centre, Harrow, UK; 3 Harrow Clinical Commissioning Group, London, UK

## Abstract

The Global Initiative for Asthma (GINA) was established in 1993 by the World Health Organization and National Heart Lung and Blood Institute to develop a global strategy for managing and preventing asthma. GINA reports, now funded independently through the sale of GINA products, have provided the foundation for many national guidelines. They are prepared by international experts from primary, secondary and tertiary care, and are annually updated following a review of evidence. In 2014, a major revision of the GINA report was published, that took into account advances in evidence not only about asthma and its treatment, but also about how to improve implementation of evidence-based recommendations in clinical practice. This paper summarises key changes relevant to primary care in the new GINA report. A noticeable difference is the report’s radically different approach, now clinically-focussed, with multiple practical tools and flow charts to improve its utility for busy frontline clinicians. Key changes in recommendations include a new, diagnosis-centred definition of asthma; more detail about how to assess current symptom control and future risk; a comprehensive approach to tailoring treatment for individual patients; expanded indications for commencing inhaled corticosteroids; new recommendations for written asthma action plans; a new chapter on diagnosis and initial treatment of patients with asthma–COPD overlap syndrome; and a revised approach to diagnosing asthma in preschool children. The 2014 GINA report (further updated in 2015) moved away from a ‘textbook’ approach to provide clinicians with up-to-date evidence about strategies to control symptoms and minimise asthma risk, in a practical, practice-centred format.

## The Global Initiative for Asthma

Asthma is a major burden worldwide, for governments, health-care providers, patients and their carers,^1^ and there is considerable variation in asthma prevalence, morbidity and mortality.^2^ Asthma remains a common cause of death in many countries,^3^ and many asthma deaths are preventable, so there is a need for a different approach.

The Global Initiative for Asthma (GINA) (www.ginasthma.org) was established by the World Health Organization and National Heart Lung and Blood Institute in 1993 to develop a global strategy for managing and preventing asthma. The GINA report is not a guideline, but a global strategy that can be adapted to local conditions; over the years, the reports have provided the foundation for many national guidelines. The GINA strategy report, prepared by international experts from primary, secondary and tertiary care, is annually updated following a review of evidence, and is now independently funded by the sale of GINA documents and resources.

Over recent years, research has led to considerable advances not only in our knowledge about asthma and treatment options^4^ but also in our understanding of how to implement evidence-based recommendations within clinical practice.^5^ This evidence is reflected in a major revision of GINA’s landmark Global Strategy Report (published in May 2014, with further minor updates in 2015).^6^ The report not only provides up-to-date evidence about strategies to control symptoms and minimise asthma risk but also presents these in a radically different style to improve their utility for busy frontline clinicians.

This paper summarises the key changes in the GINA strategy report that are most relevant to health professionals working in primary care.

## A new look for the GINA report—practical and practice-oriented

**Figure 1:**
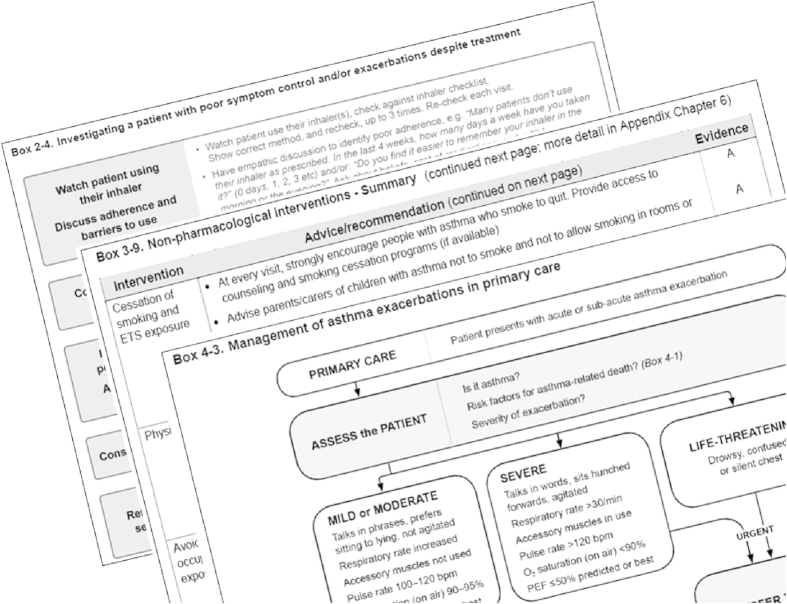
Examples of practical tools in the GINA report.

While retaining its solid evidence base, the 2014 GINA report was developed with the specific aim of improving utility for busy clinicians, with a user-friendly format, clear language and layout, and liberal use of practical summary tables and flow charts to assist with problems that clinicians face every day. These resources include flow charts for managing acute asthma in primary-care and acute-care facilities, and tables briefly summarising options for non-pharmacological treatment, and for stepping down or stepping up from different treatment regimens/doses (see Figure 1 for examples). Background detail about physiology and pharmacology was moved to an Online Appendix.^7^ Information is provided in the report not only about ‘what’ should be done and ‘why’ (what evidence supports the recommendation) but also ‘how’ it can be implemented effectively; for example, practical advice is provided about how to ask patients about their adherence in a non-judgemental way.

## What are the key content changes for primary care?

### 1. A new practical definition of asthma

‘Asthma is a heterogeneous disease, usually characterized by chronic airway inflammation. It is defined by the history of respiratory symptoms such as wheeze, shortness of breath, chest tightness and cough that vary over time and in intensity, together with variable expiratory airflow limitation’. This clinical definition, focussing on the two key features needed for the diagnosis of asthma (variable respiratory symptoms and variable airflow limitation), replaces a previous lengthy description of pathological and physiological features of asthma. For the first time, asthma is also defined as a heterogeneous disease.

### 2. Practical advice for confirming and documenting the diagnosis of asthma, to minimise under- or over-treatment

Tools include tables summarising criteria for variable expiratory airflow limitation, prioritised by reliability and feasibility for clinical practice. Clinicians are strongly encouraged to document the basis for diagnosis of asthma in individual patients; this is invaluable if the patient fails to respond to treatment or the diagnosis is in doubt. Specific advice is provided about confirming the diagnosis in special populations, e.g., the elderly, or patients presenting only with cough, and about strategies for confirming the diagnosis of asthma in patients already prescribed controller treatment.

### 3. Assess two domains of asthma control—symptom control and risk factors for adverse outcomes (also called ‘future risk’)^8^

Past asthma control assessments have focussed on current symptom control (e.g., with the Royal College of Physicians ‘three questions’,^9^ Asthma Control Test^10^ or Asthma Control Questionnaire^11^), but this is insufficient, as patients who report few symptoms may still be at risk of asthma exacerbations. Each patient’s risk factors for future exacerbations, fixed airflow limitation and side effects (also called their ‘future risk’) should also be assessed. Poor symptom control itself is a well-known risk factor for exacerbations; GINA also includes an expanded list of other risk factors that are independent of the level of symptom control, including incorrect inhaler technique, poor adherence and low lung function. A helpful table explains specific treatment for modifiable risk factors, as not all risk factors for exacerbations require a step-up in asthma treatment.

### 4. A practical algorithm for distinguishing between uncontrolled asthma and severe asthma in primary care

As above, asthma control relates both to symptom control and risk factors for future adverse outcomes such as exacerbations, and it can be quickly assessed at any time; whereas asthma severity (based on the level of treatment required to achieve good control) is a label that can only be applied retrospectively after the patient has been on treatment for at least several months. Of patients with poor symptom control and/or exacerbations despite treatment, few actually have severe refractory (i.e., treatment-resistant) asthma;^12^ the latter are estimated to comprise 5–10% of the asthma population.^13^ The GINA report provides a practical algorithm that, for primary care, prioritises the investigations for the most common remediable causes of uncontrolled asthma. It starts first with checking inhaler technique, as this is incorrect in up to 70–80% of patients^14^ and can be corrected with appropriate skills training.^15^ Confirming the diagnosis of asthma is important, as up to 25–35% of people with asthma may have been misdiagnosed;^16–19^ however, if symptoms and lung function improve substantially when inhaler technique or adherence are corrected, this may effectively confirm the diagnosis of asthma, avoiding the need for additional investigations. Patients whose asthma remains uncontrolled despite appropriate management should be referred promptly for specialist investigation and advice.

### 5. Control-based management

In the past, this concept was sometimes interpreted as prompting an automatic step-up in controller treatment if symptoms were not well controlled. Key changes for primary care in the GINA report emphasise that control-based management should include three components:

- ‘Assess’: document the patient’s symptom control and risk factors, and if these are uncontrolled, also check inhaler technique and adherence, and consider whether the symptoms are due to a co-morbid condition such as allergic rhinitis, obesity or reflux rather than asthma
- ‘Adjust treatment’ (up or down): not only drug therapy but also non-pharmacological strategies (e.g., physical activity) and treatment of modifiable risk factors (e.g., smoking cessation, providing a written asthma action plan, weight reduction). Tables with evidence levels are provided in the report
- ‘Review response’: every treatment change should be followed by a scheduled asthma review, e.g., after 2–3 months, carried out by someone with appropriate expertise, to assess and optimise control and ensure that ineffective or poorly-tolerated treatments are reviewed.

### 6. Expanded indications for starting regular controller (preventer) treatment

The GINA report now includes a new table with evidence-based recommendations for initiating controller treatment. The most important change is a recommendation for earlier initiation of low-dose inhaled corticosteroids; for example, in patients with symptoms twice or more a month and/or risk factors for exacerbations, such as a need for oral corticosteroids in the last year. The aim is to reduce asthma risk,^20–22^ even if day-to-day symptoms are not frequent enough to be burdensome.

### 7. Tailoring asthma treatment for individual patients

The GINA report now describes a framework for personalised asthma management. While national guidelines provide recommendations about the treatment that may be most cost-effective across the whole population, patient-level decisions should also take into account any characteristics or phenotypes that predict the patient’s risk or likely treatment response (e.g., smoking status, blood eosinophilia, admission to hospital for asthma in the previous year) together with patient goals and concerns and practical issues such as inhaler technique, adherence and cost to the patient.

### 8. Patients with features of both asthma and chronic obstructive pulmonary disease (COPD)

A new feature in GINA relates to the asthma–COPD overlap syndrome. Past guidelines for asthma and COPD have been separate, and most regulatory studies have excluded patients with both conditions. However, many patients have features of both asthma and COPD, including symptomatic smokers with a history of childhood asthma, and patients with asthma and fixed airflow limitation. In collaboration with the Global Initiative for Chronic Obstructive Lung Disease (www.goldcopd.org), GINA 2014 included a new chapter outlining a syndromic approach to diagnosis in primary care, with pragmatic advice about safety considerations in choice of initial treatment (avoidance of long-acting β_2_-agonist alone if the features suggest asthma, and avoiding inhaled corticosteroid-only treatment if the features suggest COPD). Therefore, it is important that asthma (or asthma–COPD overlap syndrome) should remain in the patient’s list of diagnoses, even if they develop fixed airflow limitation.

### 9. A continuum of care for worsening asthma, from early self-management, through to primary-care and acute-care management

The GINA report now includes several new tools: an evidence table summarising pharmacological options for written asthma action plans, flow charts for assessment and management of exacerbations in primary care and acute care, and a summary of key issues to be addressed during re-assessment and ongoing management after any exacerbation. Every patient should have an individualised written asthma action plan. The report provides the rationale for key new recommendations including an early increase in inhaled corticosteroid dose in written asthma action plans (unlike previous guidelines that have recommended only bronchodilator treatment and oral corticosteroids); initiation of regular inhaled corticosteroid-based treatment after any exacerbation requiring oral corticosteroids, or its resumption if the patient was previously non-adherent, and, for severe exacerbations, use of controlled-flow oxygen with a target saturation for adults of 93–95%, rather than high-flow oxygen. GINA now recommends the term ‘flare-up’ for communication with patients, as it is simpler and less ambiguous than ‘exacerbation’ or ‘attack’,^23,24^ and it reinforces the crucial message that asthma is associated with inflammation and is present even when symptoms are absent.

### 10. A new approach to diagnosing asthma in children 5 years and younger

Previous classifications of wheezing phenotypes (episodic wheeze/multiple-trigger wheeze; or transient wheeze/persistent wheeze/late-onset wheeze) have not been found to be stable, and are no longer recommended.^25^ Instead, GINA recommends that a probability-based approach should be taken to the diagnosis of asthma in preschool children, taking into account the pattern, frequency and severity of symptoms; the diagnosis should be reviewed as the child grows older. Assessment of asthma control in this age group, as in adults, should include both current symptom control and assessment of risk factors for future adverse outcomes such as exacerbations; a trial of controller therapy should be given if the symptom pattern suggests asthma, if respiratory symptoms are uncontrolled and/or if wheezing episodes are frequent or severe. The aim is to reduce the risk of future severe exacerbations, even if symptoms are currently well controlled, and to minimise the impact of uncontrolled asthma on schooling and physical and social development.^26^

## Other GINA resources

The GINA report and its Online Appendix are available for download from the GINA website (www.ginasthma.org), in the Documents and Resources tab. Also on the website are Pocket Guides summarising the key recommendations for adults/adolescents/older children and preschool children, respectively, an At-A-Glance summary, a booklet containing the chapter on asthma–COPD overlap syndrome, and a teaching slide set. Additional implementation tools, reflecting the recommendations in the GINA report, will be added during 2015. Annual updates to the GINA report will be published based on a review of recent evidence.

## Conclusion

The new GINA strategy report and supporting resources provide a substantial array of new, practical, evidence-based materials that supplement current national asthma guidelines, or can be adapted for local use, in both high- and low-resource countries. In the 2014 revision and 2015 update, GINA has moved away from a ‘textbook’ approach to provide clinicians with up-to-date evidence about strategies to control symptoms and minimise asthma risk in a practical, practice-centred format. The aim is to reduce the burden of asthma, both for patients who suffer from this disease and for health-care systems.

## Notes

HKR has been a member of advisory boards for AstraZeneca, Boehringer Ingelheim, GlaxoSmithKline and Novartis; is a member of a data safety monitoring board for AstraZeneca, GlaxoSmithKline, Merck and Novartis; has provided consulting for AstraZeneca and GlaxoSmithKline; has prepared and delivered independent medical education at symposia funded by Aerocrine, AstraZeneca, GlaxoSmithKline, Mundipharma, Novartis and Teva; and has received unrestricted research grants from AstraZeneca and GlaxoSmithKline. MLL reports personal fees and non-financial support from GlaxoSmithKline, personal fees and non-financial support from Novartis, personal fees from Clement Clarke International, personal fees and non-financial support from Teva, personal fees from AstraZeneca, non-financial support from GINA, personal fees and non-financial support from Chiesi, and personal fees and non-financial support from Consorzio Futuro In Ricerca.
